# Activity of Tricyclic Pyrrolopyrimidine Gyrase B Inhibitor against Mycobacterium abscessus

**DOI:** 10.1128/aac.00669-22

**Published:** 2022-08-25

**Authors:** Abdeldjalil Madani, Dereje A. Negatu, Abdellatif El Marrouni, Randy R. Miller, Christopher W. Boyce, Nicholas Murgolo, Christopher J. Bungard, Matthew D. Zimmerman, Véronique Dartois, Martin Gengenbacher, David B. Olsen, Thomas Dick

**Affiliations:** a Center for Discovery and Innovation, Hackensack Meridian Health, Nutley, New Jersey, USA; b Center for Innovative Drug Development and Therapeutic Trials for Africa (CDT-Africa), Addis Ababa University, Addis Ababa, Ethiopia; c Merck & Co., Inc., West Point, Pennsylvania, USA; d Department of Medical Sciences, Hackensack Meridian School of Medicine, Nutley, New Jersey, USA; e Department of Microbiology and Immunology, Georgetown University, Washington, DC, USA

**Keywords:** nontuberculous mycobacteria, NTM, SPR719, DNA gyrase

## Abstract

Tricyclic pyrrolopyrimidines (TPPs) are a new class of antibacterials inhibiting the ATPase of DNA gyrase. TPP8, a representative of this class, is active against Mycobacterium abscessus *in vitro*. Spontaneous TPP8 resistance mutations mapped to the ATPase domain of M. abscessus DNA gyrase, and the compound inhibited DNA supercoiling activity of recombinant M. abscessus enzyme. Further profiling of TPP8 in macrophage and mouse infection studies demonstrated proof-of-concept activity against M. abscessus *ex vivo* and *in vivo*.

## TEXT

Mycobacterium abscessus causes difficult-to-cure lung disease (1). Multidrug regimens are administered for months to years and typically contain an oral macrolide (clarithromycin or azithromycin) and intravenously administered amikacin, imipenem, and/or cefoxitin or tigecycline. However, cure rates are unsatisfactory, and treatment-refractory patients often undergo surgical lung resection. To further complicate treatment, the clinical utility of macrolides against M. abscessus is often limited by *erm41*-mediated inducible drug resistance (2). Given the poor performance of the current regimens, more efficacious drugs are needed. M. abscessus drug discovery efforts are hindered by extremely low hit rates in whole-cell screens attempting to identify robust chemical matter starting points (3, 4).

M. abscessus is intrinsically resistant to many antituberculosis (anti-TB) antibiotics, including all first-line drugs (5). Despite M. abscessus resistance to most approved anti-TB drugs, we found that compound collections of TB actives provide a good source for hit identification (6). Screening series of advanced TB actives against M. abscessus identified several compounds with *in vivo* activity, including inhibitors of RNA polymerase (7), ATP synthase (8), leucyl-tRNA synthetase (9, 10), DNA gyrase (11), and DNA clamp DnaN (12). Expanding on this strategy, we asked whether the recently identified novel class of tricyclic pyrrolopyrimidines (TPPs) (13), targeting DNA gyrase in Mycobacterium tuberculosis and various other bacteria (14, 15), is active against M. abscessus.

DNA gyrase is a validated drug target in mycobacteria. This type IIA DNA topoisomerase is an A_2_B_2_ heterotetrameric protein that regulates DNA topology (16). Unwinding of DNA during replication, transcription, and recombination introduces positive supercoils into the DNA molecule that, left unaddressed, impede DNA function. This problem is resolved by DNA gyrase, which introduces negative supercoils into DNA. To do this, the enzyme generates a DNA double-strand break, passes a segment of DNA through the break, and subsequently reseals the DNA molecule (16). The fluoroquinolones target the cleavage-ligation active site of DNA gyrase formed by subunits A and B, creating stalled enzyme-DNA cleavage complexes (17).

**FIG 1:**
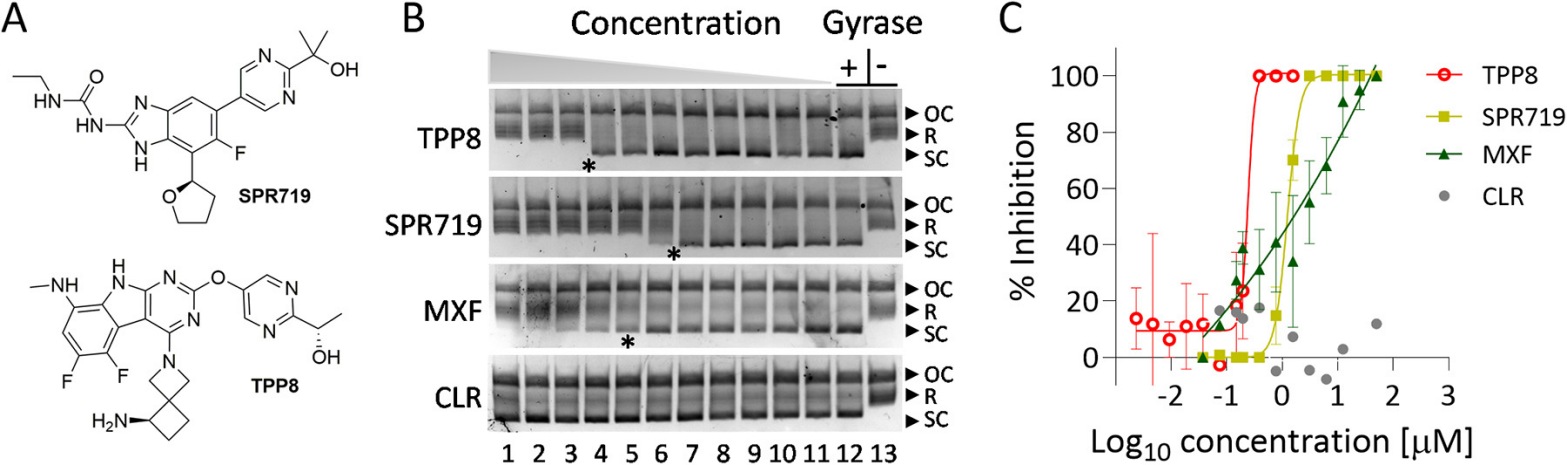
Structure and DNA gyrase inhibition activity of TPP8. (A) Structure of TPP8 and SPR719 (15, 20). (B) Effect of TPP8 and comparator compounds on the DNA supercoiling activity of recombinant M. abscessus ATCC 19977 DNA gyrase. Relaxed pBR322 plasmid was used as the substrate to measure the effect of compounds on the supercoiling activity of M. abscessus DNA gyrase as described previously (23). The conversion of relaxed (R) into supercoiled (SC) plasmid by DNA gyrase was visualized by agarose gel electrophoresis. OC, open circular plasmid. Lane 13, Gyrase -, reaction mix without added enzyme showing unaltered substrate. Lane 12, Gyrase +, reaction mix with added enzyme (without drug) showing conversion of relaxed plasmid into its supercoiled form. Lanes 1 to 11 show the effect of decreasing drug concentrations. The concentration ranges are as follows: TPP8, 1.5, 0.75, 0.37, 0.18, 0.09, 0.04, 0.02, 0.01, 0.005, 0.002, and 0.001 μM; SPR719, moxifloxacin (MXF), and clarithromycin (CLR), 50, 25, 12.5, 6.25, 3.12, 1.56, 0.78, 0.39, 0.19, 0.09, and 0.04 μM. The experiments were repeated three times independently, yielding similar results, and a representative example is shown. (C) Quantitative inhibition of DNA gyrase supercoiling activity by TPP8 and comparator drugs. The bands obtained from the three experiments represented in panel B were quantified by the Invitrogen iBright FL1000 imaging system to determine half-maximal inhibitory concentrations (IC_50_) as described previously (23). Means and standard deviations are shown. TPP8 inhibited DNA gyrase with an IC_50_ of 0.3 μM. SPR719 and MXF inhibited the enzyme with an IC_50_ of 1 μM and 3 μM, respectively (23). IC_50_ derived from panel C are indicated by asterisks in panel B. CLR, included as a negative control, did not affect the supercoiling activity of the enzyme.

Moxifloxacin is used effectively for the treatment of multidrug-resistant TB. However, the utility of this fluoroquinolone for treatment of M. abscessus infections is limited due to widespread intrinsic resistance (18). Recently, a novel benzimidazole (SPR719, Fig. 1A) entered early clinical development for mycobacterial lung diseases (19). Benzimidazoles target the ATPase domain of the DNA gyrase complex, located on its B subunits and required to drive the catalytic cycle (20), distinct from the fluoroquinolone binding site.

Similar to SPR719, TPPs were shown to bind and inhibit the ATPase domain of the gyrase B subunit in M. tuberculosis (14). To determine whether this novel class of inhibitors is active against M. abscessus, the MIC of a representative TPP compound (TPP8, compound 8 in reference 15 and in Fig. 1A [21 and 22]) (provided by Merck & Co., Inc., Kenilworth, NJ, USA) was determined. Dose-response curves were established in Middlebrook 7H9 medium using the broth microdilution method with optical density at 600 nm (OD_600_) as readout as described previously (23). TPP8 retained activity against reference strains from culture collections representing the subspecies of M. abscessus, including the type strain Mycobacterium abscessus subsp. *abscessus* ATCC 19977, and a panel of clinical isolates, including M. abscessus subsp. *abscessus* K21, used in our mouse model of infection (Table 1). With growth-inhibitory activity in the 0.02 to 0.2 μM range, TPP8 exhibited a markedly higher potency than SPR719 or moxifloxacin (Sigma-Aldrich), both showing MICs in the low micromolar range (Table 1). These results indicate that TPP8 is broadly active against the M. abscessus complex and displays potent antimycobacterial activity.

**TABLE 1:** Activity of TPP8 against M. abscessus complex

M. abscessus strain	*erm41* sequevar^*c*^	CLR susceptibility	MIC^*a*^ (μM)^*d*^			
			TPP8	SPR719	MXF	CLR
Reference strains						
M. abscessus subsp. *abscessus* ATCC 19977	T28	Resistant	0.02	1.5	3	3
M. abscessus subsp. *bolletii* CCUG50184T	T28	Resistant	0.2	1.5	3	6
M. abscessus subsp. *massiliense* CCUG48898T	Deletion	Sensitive	0.1	3	6	0.2
Clinical isolates^*b*^						
M. abscessus subsp. *abscessus*						
Bamboo	C28	Sensitive	0.2	1.5	6	0.4
K21	C28	Sensitive	0.06	1.5	3	0.2
M9	T28	Resistant	0.06	3	6	6
M199	T28	Resistant	0.02	3	3	6
M337	T28	Resistant	0.02	1.5	3	6
M404	C28	Sensitive	0.06	3	6	0.2
M421	T28	Resistant	0.06	1.5	3	3
M. abscessus subsp. *bolletii*						
M232	T28	Resistant	0.04	3	3	6
M506	C28	Sensitive	0.2	6	6	0.4
M. abscessus subsp. *massiliense* M111	Deletion	Sensitive	0.2	6	3	0.4

a MIC values are the means from three independent experiments.

b M. abscessus Bamboo (27), K21 (7), and M strains (28) were reported previously.

c *erm41*, ribosome methylase gene conferring inducible clarithromycin (CLR) resistance. “C28” and “deletion” sequevars are inactive *erm41* alleles and susceptible to CLR. The “T28” sequevar is functional and confers inducible resistance to CLR (29).

d TPP8, tricyclic pyrrolopyrimidine compound 8; SPR719, benzimidazole gyrase B ATPase inhibitor; MXF, moxifloxacin; CLR, clarithromycin (assay control).

To confirm that TPP8 exerts anti-M. abscessus whole-cell activity via inhibition of gyrase B, spontaneous resistant mutants in M. abscessus ATCC 19977 were selected on Middlebrook 7H10 agar as described previously (23). The agar MIC of TPP8 (lowest drug concentration that suppresses emergence of colonies when plating 10^4^ CFU on 7H10) was 0.64 μM as determined by the agar dilution method according to the CLSI protocol (24). To isolate spontaneous TPP8-resistant mutants, a total of 10^9^ CFU was plated on 10 30-mL agar plates containing 4 times the agar MIC, yielding one colony. TPP8 resistance was confirmed by restreaking the colony on agar containing the same TTP8 concentration. The experiment was repeated once with an independently grown culture, yielding a second TPP8-resistant M. abscessus strain. The broth MICs were similar for the two mutants, 75-fold higher than that of the wild type (Table 2). Susceptibility to moxifloxacin and clarithromycin (Sigma-Aldrich) was not affected, reducing the likelihood of a nonspecific mechanism of resistance (Table 2). Sanger sequencing of the gyrase B coding sequence, using primers GyrB-1 (GGCGTGGTGACGAGTTTAAAG), GyrB-2 (GAGATCTTCGAGACCACCACCTA), GyrB-3 (GCAAGAGTGCCACCGATATC), and GyrB-4 (GTAAGTACGACGGCACAACG) (Genewiz Inc.), showed that both resistant strains harbored a C506A (Thr169Asn) missense mutation, located in the ATPase domain (20) (Table 2). Whole-genome sequencing (Novogene Corporation Inc.) of the two resistant strains showed the absence of additional shared polymorphisms (Table 2). Interestingly, the same amino acid substitution in the M. abscessus gyrase B ATPase domain was previously shown to confer resistance to SPR719 (25). Indeed, cross-resistance studies showed that the two TPP8-resistant M. abscessus ATCC 19977 strains were resistant to SPR719 and that the previously isolated SPR719-resistant M. abscessus ATCC 19977 strain harboring the C506A missense mutation (25) was resistant to TPP8 (Table 2). To confirm that the observed missense mutation in *gyrB* indeed causes resistance, the wild type or the C506A allele of *gyrB* was overexpressed in wild-type M. abscessus ATCC 19977 using a custom-synthesized (Genewiz Inc.) pMV262-*hsp60*-based expression system for *gyrBA* as described previously (23). The strain expressing the mutant enzyme showed resistance to both TPP8 and SPR719, confirming GyrB as the intracellular target (Table 2). To directly demonstrate that TPP8 inhibits M. abscessus DNA gyrase activity, *in vitro* DNA supercoiling inhibition studies were performed using recombinant M. abscessus enzyme and plasmid pBR322 (Inspiralis) as the substrate, as described previously (23). The results demonstrate concentration-dependent enzyme inhibition by TPP8 (Fig. 1B and C). Consistent with the improved whole-cell inhibitory potency of TPP8 compared to SPR719, the compound showed higher potency against the target with a half-maximal inhibitory concentration (IC_50_) of 0.3 μM versus 1 μM for SPR719. Together, these results provide genetic and biochemical evidence that TPP8 retained DNA gyrase B as its target in M. abscessus. It is of note that the effect of TPP8 on the supercoiling activity of the resistant mutant version of the gyrase or the effect on the gyrase ATPase activity was not determined. Thus, strict biochemical proof that TPP8 acts as an inhibitor of the gyrase ATPase was not provided.

**TABLE 2:** Characterization of TPP8-resistant M. abscessus ATCC 19977

M. abscessus ATCC 19977	MIC^*a*^ (μM)^*e*^				GyrB mutation	Other mutations^*g*^	
	TPP8	SPR719	MXF	CLR		Gene	Type
Wild type	0.02	1.5	3	3	wt^*h*^		

TPP8^R^-1^*b*^	1.5	>25^*f*^	3	1.5	Thr169Asn	MAB_0568	T256C
						MAB_4048c	Del1378_1408

TPP8^R^-2^*b*^	1.5	>25^*f*^	1.5	1.5	Thr169Asn	MAB_0209	C1345A
						MAB_0209	A1384G
						MAB_0209	Ins1493CGA
						MAB_4001c	A709C

SPR^R^-L1.2^*c*^	3	>25^*f*^	3	2	Thr169Asn		

pMV262/*hsp60* empty^*d*^	0.02	1.5	3	3	wt		
pMV262/*hsp60 gyrBA*^*d*^	0.05	3.5	1.2	3	wt		
pMV262/hsp60 gyrB*A^*d*^	0.4	12.5	1.5	3	wt		

*^a^*MIC values are the means from three independent experiments.

*^b^*Independently isolated TPP8-resistant mutant strains.

*^c^*SPR719-resistant mutant strain reported previously (25).

*^d^*pMV262/*hsp60* empty, wild-type strain harboring the pMV262 expression system without gyrase genes inserted; pMV262/*hsp60 gyrBA* and pMV262/hsp60 gyrB*A, wild-type strain expressing either wild-type DNA gyrase B and A subunits or the mutant DNA gyrase B* and wild-type A subunits carried by pMV262 under the control of *hsp60* (23) with gyrase B* harboring a Thr169Asn amino acid substitution.

*^e^*TPP8, tricyclic pyrrolopyrimidine compound 8; SPR719, benzimidazole gyrase B ATPase inhibitor; MXF, moxifloxacin; CLR, clarithromycin (assay control).

*^f^*Concentrations of >25 μM could not be tested because of limited solubility of the compound (25).

*^g^*Wild type and TPP8^R^-1 and -2 were subjected to whole-genome sequencing. Shown are polymorphisms detected in the TPP8-resistant strains in addition to the mutation observed in *gyrB* via Sanger sequencing. MAB_0568, putative CarD-like transcriptional regulator; MAB_4048c, sensor-like histidine kinase senX3; MAB_0209, hypothetical protein; MAB_4001c, putative 2-nitropropane dioxygenase (https://mycobrowser.epfl.ch/).

h wt, wild type.

**FIG 2:**
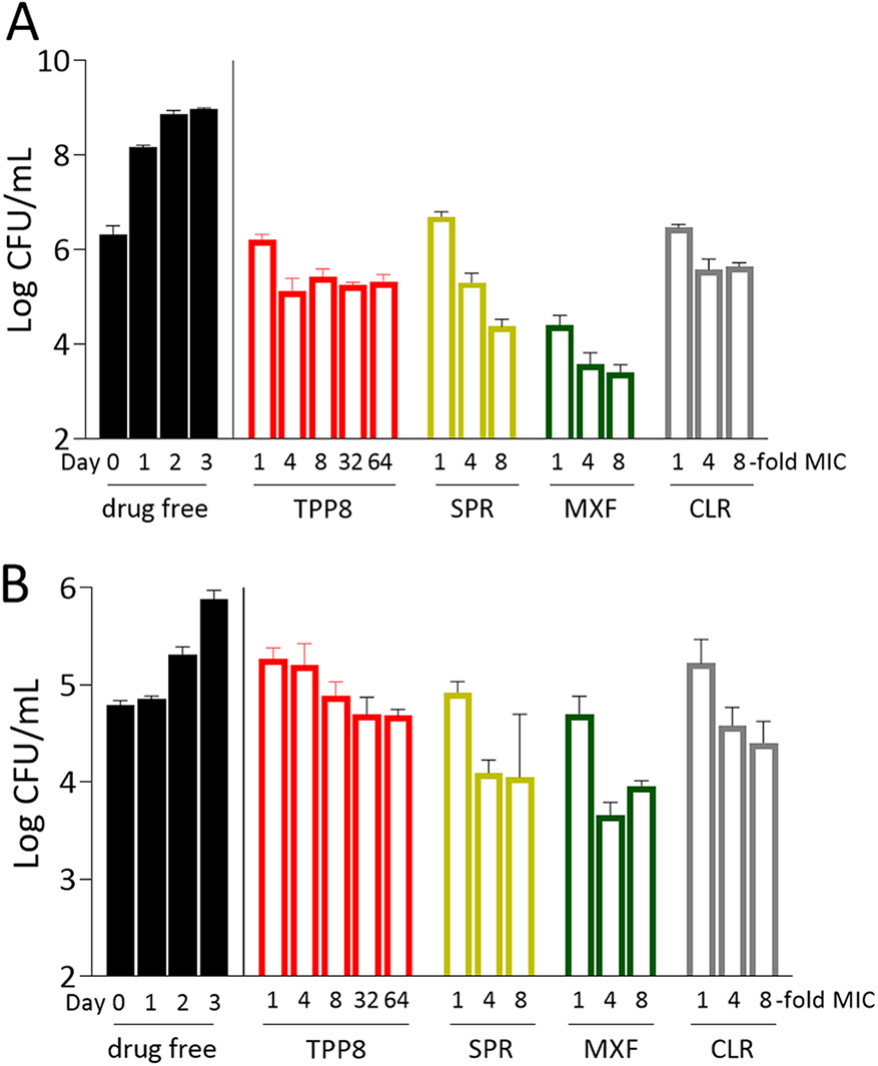
Activity of TPP8 against M. abscessus growing in broth and in THP-1-derived macrophages. (A) To determine whether TPP8 displays bactericidal activity *in vitro*, 1-mL cultures of M. abscessus ATCC 19977 growing in Middlebrook 7H9 in tubes (11) were treated with MIC multiples of TPP8, SPR719, moxifloxacin (MXF), or clarithromycin (CLR). CFU were enumerated by plating samples on Middlebrook 7H10 agar. The growth kinetics of drug-free controls are shown on the left, and the effects of TPP8 and comparators on CFU reduction are shown after 3 days of treatment. As MICs measured in tubes can be different from those measured in 96-well plates, tube MICs were measured and used as the baseline in these experiments (11). They were as follows (with MIC values shown in Table 1 and determined by the broth microdilution method in parentheses): TPP8, 0.04 μM (0.02 μM); SPR719, 6 μM (1.5 μM); MXF, 6 μM (3 μM); CLR, 1.5 μM (3 μM). (B) To determine the activity against intracellular bacteria, THP-1 cells were prepared and differentiated into macrophages with phorbol-12-myristate-13-acetate for 24 h, and the resulting macrophages were infected with a multiplicity of infection of 10 for 3 h using M. abscessus ATCC 19977 as described previously (26) and treated with the same concentration range of TPP8, SPR719, MXF, or CLR as in panel A. Intracellular CFU were enumerated by plating samples on Middlebrook 7H10 agar after 3 days of treatment. Experiments in panels A and B were carried out three times independently, and the results are represented as mean values with standard deviations.

To further characterize *in vitro* and *ex vivo* anti-M. abscessus activities of TPP8, kill experiments against M. abscessus ATCC 19977 growing in Middlebrook 7H9 broth were performed and the inhibitory potency of TPP8 against bacteria growing intracellularly in infected THP-1-derived macrophages (ATCC TIB-202) was determined (26). TPP8 was largely bacteriostatic in broth culture (Fig. 2A) and inhibited growth of intracellular bacteria (Fig. 2B). It is interesting that SPR719 exerted a weak bactericidal effect. The reason for this differential activity of the two ATPase inhibitors remains to be determined.

**FIG 3:**
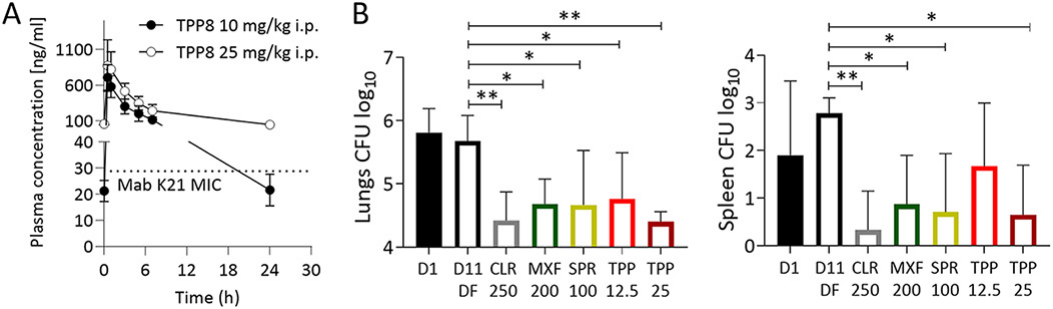
Pharmacokinetic profile and efficacy of TPP8 in mice. (A) Plasma concentration-time profile of TPP8 following a single intraperitoneal dose of 10 or 25 mg/kg in CD-1 mice. The MIC of TPP8 against M. abscessus K21 (Table 1), the strain used in our murine infection model, is indicated by a dotted line. (B) Efficacy of TPP8 and comparator compounds in a NOD SCID mouse model of M. abscessus K21 lung infection. Mouse lung and spleen CFU are shown 1 day after intranasal infection with M. abscessus K21 (D1), following daily intraperitoneal administration of 20% Solutol HS15 in phosphate-buffered saline, pH 7.4 (TPP8 vehicle), for 10 days (D11; DF, drug free), daily intraperitoneal administration of TPP8 (12.5 or 25 mg/kg), or daily oral administration of clarithromycin (CLR, 250 mg/kg formulated in 0.5% carboxymethyl cellulose), moxifloxacin (MXF, 200 mg/kg formulated in water), or SPR720 (SPR, 100 mg/kg formulated in 0.5% methylcellulose) for 10 days. Mean and standard deviation are shown for each treatment group (*n* = 6). Statistical significance of the results was analyzed by one-way analysis of variance multicomparison and Dunnett’s posttest: *, *P* < 0.01; **, *P* < 0.001. The experiment was carried out twice, and one representative data set is shown.

To determine whether the attractive *in vitro* and *ex vivo* activities of TPP8 translate into *in vivo* efficacy, an immunodeficient murine model developed by our group was utilized (7), in which mice are infected with the M. abscessus clinical isolate K21 (TPP8 MIC = 0.06 μM, Table 1) to generate a sustained infection resulting in a largely constant bacterial lung burden, thus allowing the effects of drugs to be evaluated (7). As TPP8 lacks robust oral bioavailability (15), the plasma concentration-time profile upon intraperitoneal administration in CD-1 mice (Charles River Laboratories) was determined. TPP8 plasma concentrations were measured by liquid chromatography-coupled tandem mass spectrometry. The *in vivo* pharmacokinetic analysis revealed that a dose of 25 mg/kg of body weight retains concentrations above the MIC of M. abscessus K21 for the 24-h dosing interval (Fig. 3A). Eight-week-old female NOD.CB17-Prkdc^scid^/NCrCrl mice (NOD SCID; Charles River Laboratories) were infected by intranasal delivery of 10^6^ CFU as described previously (7). TPP8 was administered intraperitoneally once daily for 10 consecutive days at 25 and 12.5 mg/kg, starting 1 day postinfection. Two comparator agents were used in the efficacy study: the phosphate prodrug form of SPR719, SPR720 (20), administered orally at 100 mg/kg, and moxifloxacin, administered orally at 200 mg/kg (11), the efficacious dose in TB mouse models (20). Clarithromycin as a positive control was administered orally at 250 mg/kg (11). All mice were euthanized 24 h after the last dose, and bacterial load in the lungs and spleen was determined by plating serial dilutions of organ homogenates on Middlebrook 7H11 agar. All experiments involving live animals were approved by the Institutional Animal Care and Use Committee of the Center for Discovery and Innovation, Hackensack Meridian Health. As expected, treatment with vehicle alone did not affect the bacterial lung burden (“D11 DF,” Fig. 3B). Compared to the vehicle control, treatment with 25 mg/kg TPP8 reduced lung CFU ~20-fold. The comparators SPR720 and moxifloxacin and the positive-control clarithromycin reduced the lung burden to a similar degree (Fig. 3B). CFU reduction in the spleen followed a similar pattern (Fig. 3B). Thus, TPP8 is efficacious in a mouse model of M. abscessus infection.

In conclusion, the tricyclic pyrrolopyrimidine TPP8 is active against M. abscessus *in vitro*, *ex vivo*, and in a mouse model of infection and exerts its antimicrobial activity by inhibiting the B subunit of DNA gyrase. This work adds a new lead compound to the preclinical M. abscessus drug pipeline and provides an attractive chemical starting point for an optimization program aiming at improving oral bioavailability. The demonstration that yet another TB active displays anti-M. abscessus activity supports the strategy of exploiting chemical matter shown to be active against M. tuberculosis to accelerate *de novo* drug discovery for M. abscessus.
